# The Emerging Portrait of Glial Cell Line-derived Neurotrophic Factor Family Receptor Alpha (GFRα) in Cancers

**DOI:** 10.7150/ijms.64133

**Published:** 2022-03-28

**Authors:** Qingshang Li, Zhijun Cao, Shuliang Zhao

**Affiliations:** Division of Gastroenterology and Hepatology, Ren Ji Hospital, School of Medicine, Shanghai Jiao Tong University; Shanghai Institute of Digestive Disease; State Key Laboratory for Oncogenes and Related Genes, Key Laboratory of Gastroenterology & Hepatology, Ministry of Health. 145 Middle Shandong Road, Shanghai, China.

**Keywords:** GFRα1, GDNF, cancer, neural invasion, treatment resistance

## Abstract

Glial cell line-derived neurotrophic factor family receptor alpha (GFRα) members have been widely connected to the mechanisms contributing to cell growth, differentiation, cell migration and tissue maturation. Here we review GFRα biological functions and discussed the evidence indicating whether GFRα signaling complex present novel opportunities for oncogenic intervention and treatment resistance. Thus, our work systematically reviewed the emerging role of GFRα family members in cancers, and provided novel insights for further researches.

## Introduction

The glial cell line-derived neurotrophic factors (GDNFs), a family of neurotrophic factors, were initially thought to be able to regulate the growth, survival, and differentiation of neural-derived cell types. However, it is becoming increasingly clear that these factors and their receptors are also widely found to express across many different cancers with further research.

The GDNF family ligands (GFLs) function through a glycosyl-phosphatidylinositol-(GPI) anchored coreceptor, GDNF family receptor alpha (GFRα), and rearranged during transfection (RET), a well-known receptor tyrosine kinase involved in kidney development, spermatogonial stem cell maintenance, and the development and maintenance of the sympathetic, parasympathetic, and enteric nervous systems 1, 2. Based on whether it cooperates with the second receptor RET, GFRα has also been widely linked to the mechanisms that contribute to cell growth, differentiation and migration and tissue maturation. However, abnormal expression or aberrant activation of these molecules may convert normal growth signals to undesirable signals inducing overgrowth, becoming an important contributor to a variety of human cancers. Importantly, increasing numbers of novel reports suggest that the GFRα-mediated signaling pathway acts as an oncogenic promoter related to tumor proliferation, invasion, and metastasis as well as treatment resistance. Thus, the role of GFRα is more complicated than originally assumed, and it is necessary to revisit and review the role played by this versatile molecule in tumors.

## GFRα Related Molecules and Signal Pathways

### Interactions of GFRα with GFLs and RET

**Figure 1:**
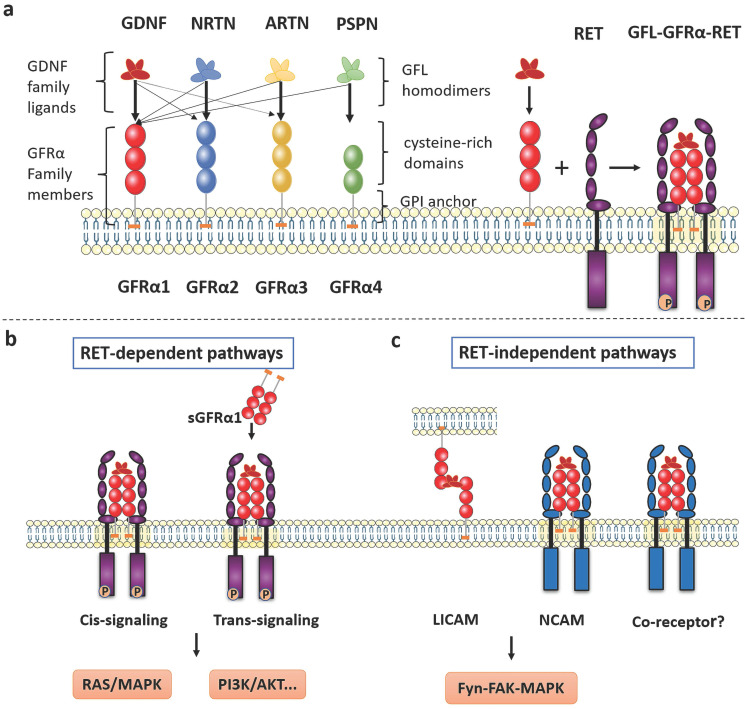
** The GFRα and GFRα-mediated signaling pathways. a** The GFRα family consists of four members, GFRα1, GFRα2, GFRα3 and GFRα4, which are tethered to the plasma membrane through GPI anchors containing CRDs. They have four characteristic ligands, namely, GDNF, NRTN, ARTN and PSPN. Two GFL monomers form an entangled homodimer to corresponding GFRα coreceptors. After GFLs and GFRα bind, the complexes associate with RET, a transmembrane tyrosine kinase coreceptor, forming a GFL-GFRα-RET ternary complex. **b** RET-dependent GFRα signaling is activated via phosphorylation of GFRα on multiple intracellular tyrosines. Two signal transduction pathways contribute to GFL-induced RET activation: via membrane-bound GFRα (cis-signaling) and soluble GFRα (trans-signaling) molecules released from nearby cells. Only the Ras/MAPK and PI3K/Akt signaling pathways are represented in the figure. **c** The presence of GDNF promotes the association of CFRα with NCAM, resulting in activation of the NCAM-mediated Fyn-FAK-MAPK signaling pathway. Other non-RET receptors of GFRα need further study. LICAM, ligand-induced cell adhesion molecules; NCAM, Neural cell adhesion molecule.

The GFRα family consists of four members, GFRα1, GFRα2, GFRα3 and GFRα4, located roughly extracellular and anchored to the plasma membrane by glycosyl-phosphatidyl-inositol (GPI). As the main component, extracellular structure contains some cysteine-rich repeats domains marked as D1-D2-D3 in GFRα1-3, and D2-D3 in GFRα4 (**Figure 1a**). Although these receptors are structurally similar, they determine specificity for four ligands—GDNF, Neurturin (NRTN), Artemin (ARTN) and Persephin (PSPN). However, the relationships among the GFLs and GFRα proteins are not strictly unique, and the ligands and receptors can cross-interact; the preferred GFRα coreceptor for GDNF is GFRα1, although GDNF also weakly binds to GFRα2 and GFRα3 3. In addition, NRTN and ARTN crosstalk with GFRα1 to activate RET. it is reported ARTN could also combine and activate both GFRα1 and GFRα3 4. PSPN not only binds GFRα4 but also signals in neurons mediated by GFRα1 5. When GFLs bind with GFRα, they form complexes and associate with the RET receptor, subsequently activating downstream signaling.

The crystal structure of GDNF was first reviewed 20 years ago 6, and other GFLs were subsequently identified 7, 8. GFLs have a relatively conserved monomeric structure consisting of an α-helical heel region, a cystine knot core motif, and pairs of antiparallel β-strand fingers. These fingers are crucial to interact with GFRα and activate RET. Currently, the two GFL monomers are thought to be arranged structurally in a “handshake”-like head-to-tail orientation to form an entangled homodimer 9, 10. On one side is a central region of GFRα comprising the D2 and D3 domains, which has been identified as a core region necessary for biochemical interaction with both GDNF and RET 11.

The RET receptor is a transmembrane tyrosine kinase with three regions: extracellular domain containing four cadherin like domains followed by cysteine rich domain, single pass transmembrane domain and tyrosine kinase domain 12. RET isoforms, which differ by 51, 43, and 9 amino acids in the C-terminus, are referred to as RET51, RET43, and RET9, respectively. The two major isoforms, RET51 and RET9, are highly conserved over a broad range of species and exert different physiological functions 13, 14. On the extracellular side of the GFRα-RET interaction, the GFL-GFRα complex associates with RET's large extracellular domain and promotes complex dimerization to form the GFL-GFRα-RET ternary complex. CLD1 and CLD2 pack together to form a clamshell-shaped structure and indirectly trap the GFL-GFRα complex, while CLD4 and CRD participate in the assembly of the signal complex 9, 15, 16. The interaction of ARTN with GFRa3 occurs through the protruding tips of fingers 1 and 2 in ARTN inserting into a pocket in the center of a triangle of a helices in the D2 domain of GFRa3, which can be described as a small hydrophobic core surrounded by a much larger halo of charged and hydrophilic interactions 17.

The consensus is that the ternary complex conforms to a stoichiometric ratio of 2:2:2 (GFL2:GFRα2:RET2) (**Figure 1a**) and that RET interacts with GFL/GFRα via two hypothetical modes 18. In the first mode, GFLs form homodimers and bind with two specific GFRα proteins, after which RET is recruited to a lipid raft membrane subdomain. After GFL-GFRα bind to each other, conformational changes in the CLD1-mediated dimerization cap facilitate RET dimerization and autophosphorylation 1, 19. In the second mode, GFRα first recruits RET to establish a preformed receptor complex that is subsequently bound by the GFL homodimer 9.

## GFRα-mediated signaling pathways

Upon interaction, RET-dependent GFRα signaling is activated via phosphorylation of RET on multiple intracellular serine and tyrosine residues, including Ser696, Tyr687, Tyr905, Tyr1015, Tyr1062, and Tyr1096 (in the RET51 isoform only), among others 1. These residues facilitate direct interactions with signaling molecules; for example, Tyr905 binds with growth factor receptor-bound protein 7/10 (GRB7/10), Tyr1015 with phospholipase C γ (PLC-γ), and Tyr1096 with GRB2‑associated binding protein 2 (GAB2) 20. Tyr1062 is the most well-characterized signaling hub for multiple adaptors containing a phosphotyrosine-binding domain (PTB) or SRC homology 2 (SH2) domain, such as fibroblast growth factor receptor substrate 2 (FRS2), downstream of kinase (DOK) family proteins (DOK1/4/5/6), and Enigma 21. Next, several well-known downstream signaling pathways are induced, including the phosphatidylinositol-3-kinase (PI3K)/protein kinase B (AKT), RAS/mitogen-activated protein kinase (MAPK), PLC-γ, and c-Jun N-terminal kinase (JNK) pathways, which lead to the survival, proliferation, differentiation, and migration of cells and potentially to oncogenesis 22. Notably, activation of RET occurs predominantly when its co-receptor GFRα bound to GFLs. Additionally, two signal transduction models contribute to GFL-induced RET activation: via membrane-bound GFRα (cis-signaling) and soluble GFRα (sGFRα, trans-signaling) molecules released from nearby cells 23, 24 (**Figure 1b**). The cis-signaling model is the classical pathway, where a cell expresses both RET and GFRα and both are stimulated in an autonomous fashion. In contrast, during activation via trans-signaling, soluble GFRα released from the membrane of neighboring cells presents GFLs to cells expressing only RET, and RET phosphorylation is then activated both inside and outside lipid rafts 22.

The differential expression of GFRα1 and RET in many tissues suggests that the presence of RET-independent pathways should pay more attention. A report indicated that GFRα1 was coimmunoprecipitated with SRC in the absence of RET suggests that GDNF signaling can pass through lipid rafts, but it is not clear how a direct interaction occurs owing to the opposite, seemingly mutually exclusive, positions of these proteins.

According to these findings, the Met tyrosine kinase receptor may be a candidate as a new transmembrane receptor to link Src with GDNF-GFRα1 25.

Neural cell adhesion molecule (NCAM) is a homophilic binding glycoprotein playing critical roles in cell-cell adhesion, neurite outgrowth, and synaptic plasticity 26. Interestingly, GFRα, as a coreceptor for GDNF, interferes with NCAM function by silencing NCAM homophilic interactions and NCAM-mediated cell adhesion 27 (**Figure 1c**). When GDNF is lacking, GFRα inhibits NCAM-NCAM interactions as a negative regulator (short-range). By contrast, the presence of GDNF promotes the association of CFRα and NCAM, resulting in activation of the NCAM-mediated Fyn-FAK-MAPK signaling pathway (long-range) 23. Regarding cell adhesion molecules, GDNF can induce the association of membrane-bound GFRα from non-same cells (trans-homophilic interactions), allowing interaction between neuronal and glial cells. Therefore, a new role for GFRα proteins can be described, in which these proteins act as ligand-induced cell adhesion molecules (LICAMs) that influence extracellular crosstalk 28 (**Figure 1c**).

## GFRα-induced Oncogenesis

### Breast cancer

GFRα1 expression is upregulated in a significant proportion of human breast cancers 29-31. Abundant expression of GFRα1 was confirmed in tissues of luminal A breast cancer, which comprise 70% of breast cancer cases, while minimal or no expression was observed in normal human breast tissue. Expression of GFRα1 or GFRα3, particularly bound with ARTN, has been consistently associated with poor survival outcomes, so these proteins can serve as prognostic markers in specific subtypes of mammary carcinoma 32. In recent, a positive feedback loop was demonstrated between a GFRα1 and a certain gene. On the one hand, GFRα1 was identified as a target protein of ST3 beta-galactoside alpha-2,3-sialyltransferase 1 (ST3GAL1), which regulates the GDNF/GFRα1/RET pathway in breast cancer cells by mediating O-linked sialylation of GFRα1 and facilitating its interaction with RET. On the other hand, GFRα1-mediated signaling was found to stimulate the transcription of ST3GAL1 through the AKT/Sp1 pathway 33. In addition, inhibition of the RET receptor decreases the growth and metastatic potential of ER+ breast cancer cells. In other words, GDNF-mediated GFR/RET activation promotes breast cancer proliferation and migration. Mechanistically, this activation might rescue cells from the antiproliferative effects of endocrine therapy and stimulate the expression of cytokines, especially the inflammatory cytokine IL6 34. Although RET is extensively involved in the development of breast cancer, GFRα is indispensable and irreplaceable in driving endocrine resistance, thus contributing to cell survival 35. Moreover, anti-GFRα1 antibodies display robust therapeutic activity in clinically relevant cell line-derived xenograft models 36. Therefore, high expression of GFRα1 is associated with poor prognosis in patients with high-grade breast cancers 37, 38.

### Osteosarcoma

The GFRα1-dependent pathway has often been related to treatment resistance in tumors. After treating osteosarcoma cells with cisplatin, a widely used anticancer drug, it induces the overexpression of GFRα1, which promotes autophagy to lead to enhanced osteosarcoma cell survival via the SRC-AMPK signaling axis. Moreover, GFRα1 is involved in chemoresistance in osteosarcoma independent of RET and its major ligand GDNF, as confirmed by Mihwa Kim 39, 40. These investigations suggest that GFRα1 could be a therapeutic target for the prevention of chemoresistance in osteosarcoma.

### Pancreatic cancer

Recently, the importance of the PC-promoting role of GFLs and GFRα has become more prominent and better understood. The expression of GFRα and GFLs is barely detectable in normal pancreatic tissues, but both are upregulated overall in PC 41, 42. Increased NRTN/GFRα-2 levels in PC promote an aggressive pancreatic cancer cell (PCC) phenotype, enhancing PC invasiveness. In addition, GFRα-2 but not NRTN is associated with the sensation of severe abdominal pain in PC patients 42. The mechanism may be related to the transmission of neural signals.

GFRα1/RET receptor complex promotes the proliferation and invasion of PCC by binding to GDNF in an autocrine/paracrine manner 43. The results of adhesion and invasion assays revealed that the enhanced expression and associated increase in the adhesive and invasive abilities of PCCs were inhibited by GFRα1 blockade 44. Apurinic/apyrimidinic endonuclease 1 (Ape1/Ref-1)-induced GFRα1 protein expression via nuclear factor kappa B (NF-κB) contributes to GDNF-induced Matrix metalloproteinase-9 (MMP-9) expression, which strongly correlates with the desmoplastic reaction and lymphoid invasion; this mechanism might partially underlie the invasive behavior of PCCs 45. In the tumor microenvironment, GFRα1 was demonstrated to be released by nerves, enhancing perineural invasion (PNI) and serving as a guidance signal for cancer cell migration. Notably, GDNF expression, RET phosphorylation, and MAPK pathway activity were found to be increased in a dose-dependent manner after exposure to soluble GFRα1 46.

Both ARTN and its receptor complex GFRα3/RET were found to be overexpressed in PC, not only in primary cancer cells but also the surrounding tissues 47. These mediators can promote the motility and invasiveness of MIA PaCa-2 cells. When ARTN treatment was administered, MMP-2 expression increases, and E-cadherin expression decreases 48. Most notably, ARTN/GFRα3 increases the migration and invasion of PCCs in a manner like GDNF/GFRα1 42, 47.

### Prostate cancer

GDNF and GFR α 1 are secreted by the increased nerves in the peritumoral stroma of prostate cancer to create a perineural niche where RET signaling can occur. These factors are secreted via paracrine signaling, and some prostate cell lines can also express and specifically secrete GFRα1, perhaps via an autocrine mechanism 49. In prostate cancer, GFRa1 plays a limiting role that supports GDNF/RET signaling to activate both the PI3K/AKT and MAPK/ERK pathways through phosphorylation of RET on Tyr1062, enhancing proliferation *in vitro* and tumor growth *in vivo* 1, 49. Furthermore, GDNF stimulation increased the proliferation rate of prostate cancer cells and activated the signal pathway through GFRα1/SRC pathway, which was related to the expression level of GFRα1, but not related to RET. In addition, GFRα1/SRC activation can promote homing of resistant prostate cancer cells to a microenvironment with augmented growth-promoting and resistance-inducing properties 50. Despite a report indicating coimmunoprecipitation of GFRα1 and SRC, whether they interact directly needs further verification due to their positions on opposite sides of the lipid bilayer.

### Neuroblastoma

GFRα2 is upregulated in neuroblastoma cells and tissues, and its overexpression promotes neuroblastoma cell proliferation. As revealed by a recent study using colony formation assays and western blot analysis, GFRα2 interacts with phosphatase and tensin homolog (PTEN), a tumor suppressor that inhibits the well-known PI3K/AKT pathway. Consequently, GFRα2 promotes neuroblastoma cell proliferation by activating the PI3K/AKT pathway 51. GFRα1 is a direct target of Ape1/Ref-1 in Neuro2a mouse neuroblastoma cells. Ape1/Ref-1 expression causes the clustering of GFRα1 in lipid rafts in response to GDNF, contributing to phosphorylation of AKT and PLCγ-1 and stimulating cell proliferation 52. Another report 53 showed that the inhibitor of PLC-γ blocks the pro-survival effect of GDNF on the spinal motoneurons *in vitro*, but it's an indirect data. There are several studies indicating that GDNF may activate PLC-γ signaling pathway, but additional work is needed to answer this question.

### Colorectal cancer

GDNF and NRTN were highly expressed in colorectal cells, whereas the coreceptor GFRα1 and RET were expressed in the surrounding ganglia and glial cells 54. Increased expression of GDNF enhances β1 integrin expression via signaling through RET/GFRα1 in colorectal cancer cell lines, thus strongly influencing adhesion to and invasion of the extracellular matrix (ECM). Subsequently, these cancer cells exhibit increased invasive ability and malignancy 55. According to a recent report, demethylation of *GFRα1* is a frequent event during colorectal cancer development, and high dm*GFRα1* levels can result in GFRA1 overexpression and significantly increase cancer malignancy 56; similar results were also observed in gastric cancer 57. Further research showed that GFRα1 enhances proliferation probably by activating the AKT and ERK pathways; thus, GFRα1 might be a marker for poor prognosis in colorectal cancer 56.

### Gastric cancer

In addition, genome-wide DNA methylation analysis showed that methylation changes in *GFRα1* are positively correlated with gastric carcinoma metastasis 57. Similarly, the *GFRα3* promoter region was shown to be markedly hypermethylated in almost all gastric tumors 58. However, whether these changes can strongly influence the relevant phenotypes is less clear.

### Lung cancer

ARTN, RET, and GFRα3 have been demonstrated to be upregulated in non-small cell lung carcinoma (NSCLC) cells compared with their normal counterparts, while high ARTN expression also enhances the migration and invasion of NSCLC cells. The oncogenic effect of ARTN is correlated with BCL2 expression, and these two phenomena may be causally related. Notably, both GFRα3 and GFRα1 are expressed in H1299 cells, whereas GFRA3 is expressed only in H1975 cells 59.

### Other cancers

In acute myeloid leukemia cells, RET signaling was observed to be activated via ARTN/GFRα3 and NRTN/GFRα2 ligand/coreceptor complexes, and mTORC1-mediated suppression of autophagy was identified as a downstream pathway 60. The differential activity of GFRα pathways in different cancers are shown in **Table 1.** More details on study methods or antibody specificity of above reviewed literatures are listed in Supplementary Table 1.

## GFRα and neural invasion

Tumor invasion and migration are major reasons for poor prognosis and are frequently the cause of cancer-related deaths. These unfavorable behaviors are closely associated with the interaction between tumor cells and the tumor microenvironment. The most remarkable role of GFL-GFRα signaling in cancers is modulating the relationship between the tumor and its surroundings. Indeed, the interaction between the two can form a reciprocal loop, leading to enhanced tumor cell malignancy.

Cancer spreads via three classical mechanisms: direct invasion of surrounding tissue, lymphatic spread and hematogenous spread. However, a fourth route of spreading, neural dissemination, should be highlighted. The presence of PNI is a key feature most strongly associated with poor prognosis and high recurrence in colorectal cancer, gastric cancer, oral squamous cell carcinoma (OSCC), and pancreatic cancer 61.

GFRα serves as a coreceptor with RET on the surface of cancer cells to activate downstream signaling, cancer cell migration, and PNI. During PNI, a soluble form of GFRα1 released by normal nerves facilitates neural tracking regardless of GFRα1 expression in cancer cells 62. Migration of human pancreatic adenocarcinoma MiaPaCa-2 cells toward nerve-secreted GDNF, phosphorylation of RET, and MAPK pathway activity are increased dose-dependently upon exposure to soluble GFRα1. Even though GFRα1 expression varies widely in different cancer cells, both GFRα1 and its ligand GDNF can be released from the tumor microenvironment and cooperate to facilitate cancer invasion 46. According to another report, the expression of RET and GRFα1 is higher in tumor tissues of patients with neuroinvasive pancreatic carcinoma than in normal tissues. In an *in vitro* Matrigel coculture model of dorsal root ganglion and PCCs, nerve-secreted GDNF induced polarized neurotrophic migration of cancer cells (PNMCs) along the nerve axons, whereas deficiency of this mediator reduced the ability to attract cancer cells. Potentially, the MAPK pathway might be stimulated by GDNF-GFRα1-RET signaling to mediate nerve invasion 63. Accordingly, systemic therapy with pyrazolopyrimidine-1, a tyrosine kinase inhibitor targeting RET, suppresses and abolishes nerve invasion toward the spinal cord and prevents further damage 63, 64. Via an alternative pathway, increased expression of NRTN and GFRα2 by cancer cells has been linked to nerve invasion and severe pain, indicating a poor prognosis for these patients with pancreatic ductal adenocarcinoma 42. Moreover, in *in vivo* and *in vitro* experiments, overexpression of ARTN not only promoted the proliferation of PCCs but also enhanced their ability to invade peripheral organs, nerves and lymph nodes 65.

PNI is another prominent characteristic of head and neck cancers, occurring in as many as 5-90% of patients 66. GDNF-increased cancer cell aggressive behavior was markedly reduced in oral cancer when pharmacological inhibitors or neutralizing antibodies inhibited MMP-9 (matrix metalloprotein 9) and MMP-13, an enzyme family destroying the histological barrier of tumor cell invasion. Further protein assays revealed that GDNF also increases ERK, p38 and JNK phosphorylation and AP-1 DNA binding activity to facilitate the interactive invasion and growth of cancer cells and nerves 67. Similarly, in colorectal cancer cell lines, β1 integrin expression is enhanced by increased GDNF expression via signaling through RET/GFRα1, notably influencing adhesion to and invasion of the ECM. Consequently, these cancer cells exhibit increased invasive ability and malignancy 55.

## GFRα and Treatment Resistance

### Chemoresistance

**Figure 2:**
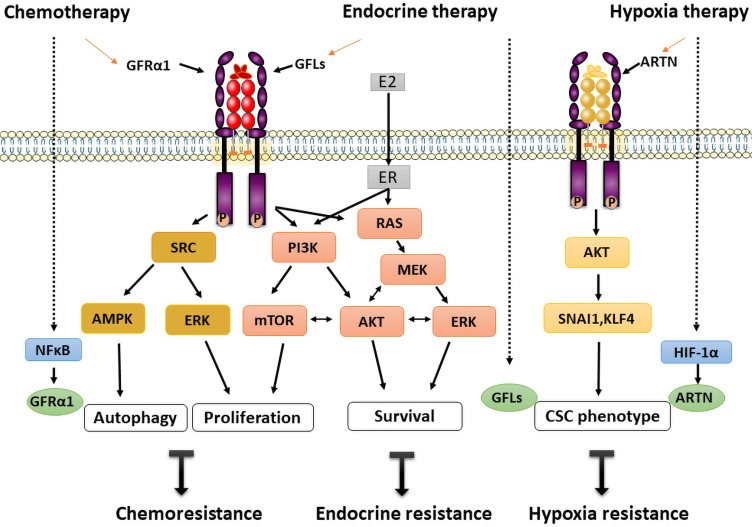
** GFRα and treatment resistance. Chemoresistance:** Cisplatin stimulates overexpression of GFRα1 via NFκB phosphorylation and decreases cisplatin-induced apoptosis, accompanied by increased autophagy, and significantly promotes cell proliferation through the SRC-AMPK signaling axis. **Endocrine resistance:** GFLs/GFRα/RET and ER signaling participate in intricate crosstalk via the PI3K/mTOR and RAS/MEK/ERK pathways in breast cancer. Endocrine therapy promotes the expression of GFLs, resulting in a vicious loop of RET signaling. **Hypoxia resistance:** Hypoxia directly activates ARTN transcription via HIF-1α, and the ARTN-dependent AKT pathway is then activated to trigger expansion of the CSC population. The solid arrows indicate the known and direct interactions between signaling molecules; the broken arrows indicate interactions requiring further investigation. The red arrows indicate the main GFRα signaling pathway.

Autophagy is a self-eating mechanism to maintain cellular homeostasis in cell survival in adverse environments, such as those established by irradiation, cytotoxicity and hypoxia 68. GFRα1-induced cancer cell autophagy is a recently identified novel regulatory mechanism of osteosarcoma chemoresistance. In two osteosarcoma cell lines, MG-63 and U-2 OS, GFRα1 expression was upregulated at both the transcriptional and translational levels following treatment with cisplatin, as evaluated by measuring the expression and phosphorylation levels of NFκB. Overexpression of GFRα1 decreased cisplatin-induced apoptosis, accompanied by increased autophagy, and significantly promoted cell proliferation. Further molecular studies showed that GFRα1 overexpression is mediated through the SRC-AMPK signaling axis and enhances the expression of downstream molecules including beclin1, etc. The results of animal experiments confirmed that the mechanism by which cancer cells survive through chemical resistance may be GFR 1-mediated autophagy 39, 40 (**Figure 2**).

Similarly, transcription of GDNF in PSC27 prostate cancer cells was found to increase by several fold following exposure to cytotoxic agents. DNA damage caused by those drugs induced abundant GDNF secretion from cells in the tumor microenvironment, which then stimulated the growth of stromal cells and prostate cancer cells through an autocrine/paracrine loop via the SRC/ERK pathway. Additionally, tumor cells become resistant to mitoxantrone and docetaxel chemotherapy, which leads to acquired treatment resistance and can be induced by exposure to GDNF. Further gene analysis indicated that overexpression of *RET* and *GFRα1* could be considered to act via a GDNF coreceptor to increase the mitotic rate. Moreover, only *GFRα1* expression correlates with migration and invasion of prostate cancer, not *RET* and *GFRα2-4* 50. In summary, based on the balance of autophagy and selective proliferation, re-proliferation of drug-resistant tumor cells is suggested as a mechanism underlying rapid tumor recurrence and treatment failure.

### Endocrine resistance

ER+ subtypes account for the majority of breast cancers and have exhibited good outcomes after endocrine therapy, which has been a first-line treatment for decades. Many patients exhibited a survival benefit of significantly longer survival times. However, GDNF-GFRα1-RET signaling is decisive in endocrine therapy resistance in ER+ breast cancers.

In an *in vitro* model of MCF7 cells, GDNF-mediated signaling was enhanced and promoted the survival of aromatase inhibitor-resistant cells. However, this increased resistance was selectively reversed by the RET kinase inhibitor NVP-BBT594. Moreover, gene analysis indicated that a GDNF response gene set predicts poor prognosis and has predictive value in breast cancer 38. Further study showed that endogenous GDNF can be produced by endocrine-resistant cells and can be secreted into the medium and activate GFRα1/RET signaling in nearby cells 35. Other RET ligands, ARTN and NRTN, but not PSPN, can also initiate and confer endocrine resistance. For instance, acquired tamoxifen resistance was induced by an estrogen-regulated gene, ARTN, which mediated increase of BCL-2 expression and promoted radioresistance and chemoresistance by enhancing cancer stem cell (CSC)-like behavior in breast cancer cells 69, 70. Furthermore, ARTN depletion unexpectedly reversed trastuzumab sensitivity, resulting in trastuzumab resistance in HER2-positive cells 71.

Estrogen receptor signaling pathway plays a critical role in the occurrence and development of breast cancer. When GDNF was applied to MCF7 cells as a model of ER+/GFRa1+/RET+ breast cancer, RET signaling resulted in increased ER phosphorylation predominantly via the mTOR pathway and estrogen-independent transcriptional activation of ER-dependent genes 31. RET downstream signaling leads to ER phosphorylation through mTOR independent of PI3/AKT and via a possible compensatory mechanism through the MEK-ERK pathways 31. The interaction of GFLs/GFRα/RET and ER signaling establishes an intricate crosstalk network in breast cancer. Another interesting hypothesis is that estrogen-induced upregulation of ARTN and GDNF promotes tumorigenesis, which leads to activation of RET-related signaling, a vicious circle 31 (**Figure 2**).

Collectively, ER+ breast cancer cells may be “poised” for GFRα/RET-mediated endocrine resistance 35. However, because the understanding of this unfavorable phenomenon is gradually increasing, novel corresponding targeted treatments are rapidly emerging.

### Hypoxia resistance

Hypoxia, a major feature of solid tumors, commonly develops owing to dramatic cell proliferation and inadequate blood supply, which increases patient treatment resistance and favors tumor progression 72. Recently, accumulating evidence has indicated that ARTN is closely associated with a higher clinical stage and poor prognosis of hepatocellular carcinoma (HCC) patients. ARTN was shown to enhance the tumorigenicity of HCC cells *in vitro* by reducing apoptosis and increasing epithelial-mesenchymal transition (EMT) and *in vivo* by promoting xenograft tumor growth and metastasis. Moreover, hypoxia directly activates ARTN transcription via hypoxia-inducible factor-1α (HIF-1α), and the ARTN-dependent AKT pathway is then activated to induce expansion of the CSC population. A novel HIF-1α/ARTN/AKT axis mediating hypoxia-induced EMT and CSC promotion in HCC cells is thus formed (**Figure 2**). Herein, ARTN is considered not only a hypoxia-responsive factor but also an indispensable factor for hypoxia-induced cell expansion in HCC 73. Via this mechanism, ARTN facilitates cancer cell evasion of hypoxia-related therapies and is thus a valuable potential therapeutic target.

## Conclusions

Herein, we reviewed GFRα biology and physiology and discussed the evidence indicating whether GFRα signaling complex present novel opportunities for oncogenic intervention. The GFRα family constitutes a group of four structurally related receptors that have historically been regarded to play developmental roles in the kidney and neuronal system. More recently, however, they have been credited with additional developmental functions during cancer progression. A literature review indicated that the GFRα family, consisting of GFRα1-4, is involved in breast cancer, colorectal cancer, prostate cancer, lung cancer, gastric cancer, and many other tumors, thus exhibiting a diverse oncogenic portfolio. Additionally, GFRα is prominently involved in mediating tumor peripheral infiltration and treatment resistance.

However, many questions about the role of GFRα1 signaling in tumor progression need to be studied and resolved. For example, 1) what are the regulatory factors and mechanisms underlying the differential expression of GFRα in tumors of different tissue types? 2) Are additional unrecognized coreceptors, interacting proteins or crosstalk pathways involved in GFRα signaling? 3) What is the clinical effect of GFRα1 as a therapeutic target in different tumors? Looking forward, a further understanding of the mechanisms involving GFRα family members may provide critical strategies toward the discovery of novel potential approaches for long-term tumor treatment.

## Supplementary Material

Supplementary table.

## Figures and Tables

**Table 1:** Differential activity of GFRα pathways in different cancers

Cancer	Ligands	Receptors	Primary pathways	Main effects	Refs
Breast cancer	GDNF	GFRα1/RET	PI3K/AKT, ST3GAL1	Proliferation	33
			FAK/STAT	Migration	34
			ERK	Endocrine therapy resistance	35
	ARTN	GFRα1, GFRα3	-	Worse survival outcome	32
Osteosarcoma	-	GFRα1	SRC-AMPK, NFκB	Autophagy and chemoresistance	39, 40
Pancreatic cancer	NRTN	GFRα2	-	Severe cancer pain and neuroplasticity	42
	GDNF	GFRα1	Ape1/Ref-1, MMP-9	Invasion	45
		soluble GFRα1/RET	MAPK/ERK	Perineural invasion	46
	ARTN	GFRα3/RET	MMP-2, ECM	Invasiveness	48
Prostate cancer	GDNF	GFRα1/RET	PI3K/AKT, MAPK/ERK	Proliferation and invasion	49
	GDNF	GFRα1	SRC/ERK	Proliferation and treatment resistance	50
Neuroblastoma	-	GFRα2	PI3K/AKT, PTEN	Proliferation	51
	GDNF	GFRα1	PLCγ-1, AKT, Ape1/Ref-1		52
Colorectal cancer	GDNF	GFRα1/RET	RAS/PI3K/AKT RAS-RAF1-MEK1/2-ERK1/2	Proliferation and survival	56
Lung cancer	ARTN	GFRα3/RET	Bcl-2	Proliferation and invasion	59
Acute myeloid leukemia	NRTN	GFRα2	mTORC1	Autophagy suppression	60
	ARTN	GFRα3			

PI3K, phosphatidylinositol 3 kinase; AKT, protein kinase B; FAK, focal adhesion kinase; STAT signal transducer and activator of transcription; ERK, extracellular-signal-regulated kinase; SRC, AMPK, Adenosine 5'-monophosphate (AMP)-activated protein kinase; NFкB, nuclear factor-kappa beta; Ape1/Ref-1, apurinic/apyrimidinic endonuclease 1; MMP-9, matrix metalloproteinases-9; MAPK, mitogen activate protein kinase; ECM, extracellular matrix proteins; PLC-γ, phospholipase C γ; MEK, mitogen-activated protein kinase kinase.
